# Seasonal variability of mercury concentration in soils, buds and leaves of *Acer platanoides* and *Tilia platyphyllos* in central Poland

**DOI:** 10.1007/s11356-016-6179-2

**Published:** 2016-02-04

**Authors:** Artur Kowalski, Marcin Frankowski

**Affiliations:** Department of Water and Soil Analysis, Adam Mickiewicz University in Poznań, Umultowska 89b, 61-614 Poznań, Poland

**Keywords:** Mercury, Soil, *Acer platanoides*, *Tilia platyphyllos*, CV-AFS

## Abstract

In this paper, we present the results of mercury concentration in soils, buds and leaves of maple (*Acer platanoides*—*Ap*) and linden (*Tilia platyphyllos*—*Tp*) collected in four periods of the growing season of trees, i.e. in April (IV), June (VI), August (VIII) and November (IX) in 2013, from the area of Poznań city (Poland). The highest average concentration of mercury for 88 samples was determined in soils and it equaled 65.8 ± 41.7 ng g^−1^ (range 14.5–238.9 ng g^−1^); lower average concentration was found in *Ap* samples (*n* = 66): 55.4 ± 18.1 ng g^−1^ (range 26.5–106.9 ng g^−1^); in *Tp* samples 50.4 ± 15.8 ng g^−1^ (range 23.1–88.7 ng g^−1^) and in 22 samples of *Tp* buds 40.8 ± 22.7 ng g^−1^ (range 12.4–98.7 ng g^−1^) and *Ap* buds 28.2 ± 13.6 ng g^−1^ (range 8.0–59.5 ng g^−1^). Based on the obtained results, it was observed that the highest concentration of mercury in soils occurred in the centre of Poznań city (95.5 ± 39.1 ng g^−1^), and it was two times higher than the concentration of mercury in other parts of the city. Similar dependencies were not observed for the leaf samples of *Ap* and *Tp*. It was found that mercury concentrations in the soil and leaves of maple and linden were different depending on the period of the growing season (April to November). Mercury content in the examined samples was higher in the first two research periods (April IV, June VI), and then, in the following periods, the accumulation of mercury decreased both in soil and leaf samples of the two tree species. There was no correlation found between mercury concentration in leaves and mercury concentration in soils during the four research periods (April–November). When considering the transfer coefficient, it was observed that the main source of mercury in leaves is the mercury coming from the atmosphere.

## Introduction

In the natural environment, mercury constantly undergoes complex transformations at different levels of its cycle in the air, water, soil, bottom sediments, plants and living organisms (Horvat et al. 1999; Melamed and Villas Bôas 2000; Ericksen and Gustin 2004; Brent and Berberich 2014; Looi et al. 2015; Ma et al. 2015). Owing to its toxicity, mobility and long residence time in the atmosphere, mercury is considered as one of the major hazardous substances (Poissant et al. 2008; Wang et al. 2012; Pérez-Sanz et al. 2012; Rallo et al. 2014; Siudek et al. 2015). Prior to its re-deposition on the surface of the land or ocean, mercury can be retained in the atmosphere for a period of 6 to 24 months. Such pollutants as mercury can be transported thousands of kilometres from the emission source, causing regional and global pollution problems (Wang et al. 2012; De Simone et al. 2014; Jang et al. 2014; Wang et al. 2014b, Chételat et al. 2015). Therefore, there is a constant need to monitor mercury-related processes in order to understand the biogeochemical cycle of this element in the environment (Hellings et al. 2013; Lodenius 2013). There are two ways of mercury transfer to plants: from the atmosphere and from the soil (Kabata-Pendias 2001; Ericksen and Gustin 2004; Poissant et al. 2008, Pérez-Sanz et al. 2012). The availability of mercury in soil is low for plants, and its accumulation in roots is not observed. The roots act as a barrier against mercury uptake. This was found in the studies of polluted soils, in which a positive correlation between mercury concentrations in the soil and plants was not determined (Pérez-Sanz et al. 2012; Lodenius 2013; Amorós et al. 2014). It has been reported that the main source of mercury in leaves is the atmosphere (Ericksen et al. 2003; Fay and Gustin 2007; Windham-Myers et al. 2014). Transfer of mercury (gaseous and elemental mercury forms) from the atmosphere occurs by dry deposition and wet deposition (rain and snow) (Fay and Gustin 2007; Poissant et al. 2008; Adjorlolo-Gasokpoh et al. 2012; Niu et al. 2013; Lodenius 2013). It was found that mercury concentration in leaves varies with the age of a plant, time of day and year (Ericksen et al. 2003; Rutter et al. 2011; Tabatchnick et al. 2012). High concentration of mercury in plants can cause negative biochemical effects such as changes in cell membrane permeability, inhibition of protein synthesis, bonding to sulphuryl groups, interference in photosynthetic and evaporation processes, and development of chlorosis (Kabata-Pendias 2001; Wang et al. 2012; Lodenius 2013). Moreover, the trees growing in large urban areas and experiencing the adverse effects of mercury may develop conditions such as chlorotic spots, brown spots on the edges of the lamina, and shortening and deformation of buds and roots. It has been estimated that the average content of mercury in plants is 100–1000 ng g^−1^ (Yudovich and Ketris 2005; Garcia-Sánchez et al. 2009). Mercury is adsorbed on colloids in soil, and the rate of this process depends primarily on the composition of the soil, amount of organic matter, quantity and type of clay minerals, hydrated oxides, pH and redox potential (Kabata-Pendias 2001; Pérez-Sanz et al. 2012; Zhang et al. 2015). Mercury in soil originates from three sources:

1. Natural processes, i.e. weathering of rocks, volcanic eruptions and geothermal activity.
2. Anthropogenic activities and re-deposition of mercury previously emitted to the atmosphere (Wang et al., 2012; Wang et al. 2014a); it is believed that deposition from the atmosphere is the main anthropogenic source of mercury in soils (Gupta and Nirwan 2015).
3. Leaves fallen from trees; they supply surface soil layers with mercury (Ericksen et al. 2003; Rutter et al. 2011; Tabatchnick et al. 2012).

Organic matter from the fallen leaves is mineralized, and mercury is released into the soil (Tabatchnick et al. 2012; Wang et al. 2012; Hellings et al. 2013; Rutter et al. 2011). Soils from treeless areas contain less mercury than those from woodland areas (Ericksen et al. 2003). In dry soils, mercury bound to organic matter can be methylated by bacteria and become a source of methylmercury both for the soil and water (Kloke et al. 1984; Rutter et al. 2011). Determination of natural mercury content in soils is difficult. This value has been estimated to be between 20 and 190 ng g^−1^ (Li and Wu 1991; Curlic et al. 1995; Chen et al. 1999; Brabo et al. 2003; Wang et al. 2012), while the average content of mercury in soils of different world regions is in the range of 50–500 ng g^−1^ (Kabata-Pendias 2001; Wang et al. 2012). Many authors have studied the effect of mercury concentration on the soil and air on the content of this metal in plants, especially in their leaves (Ericksen et al. 2003; Ericksen and Gustin 2004; Fay and Gustin 2007; Niu et al. 2013; Amorós et al. 2014). However, in most cases, the studies were carried out in laboratory conditions. They were focused on assessing changes in the mercury content of plants during the experiment in which mercury concentrations in the soil or air were modified. Young or annual plants were most often used in the experiments, as they are more susceptible to pollution than older or perennial plants.

The aims of this study were to (1) determine total mercury content in the samples of soils and plants (buds and leaves) of two tree species: *Acer platanoides* (*Ap*) and *Tilia platyphyllos* (*Tp*); (2) present spatial distribution of mercury in the soil and plants of the Poznań area; (3) estimate the variability of mercury concentration in plants during different periods of the growing season: April (IV), June (VI), August (VIII) and November (XI); and (4) determine the relationship between mercury concentrations in soil samples and the two tree species, by using statistical tests.

## Materials and methods

### Study area

**Fig. 1:**
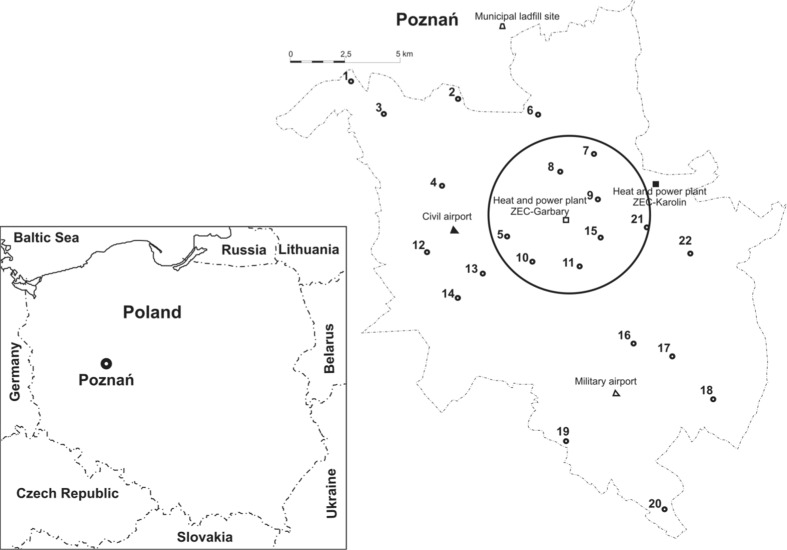
Map of the study area with sampling points and potential emission sources. Points located in the city centre are in the *circle*

The city of Poznań (area: 261.8 km^2^, population: ~600 000) is located in the Wielkopolska province, in central Poland (Fig. 1). The city is a central part of the Poznań Agglomeration, bordering Luboń and Swarzędz towns and 11 municipalities. Poznań has a direct access to five national roads and, on the south, to A2 motorway. The city has two airports: civilian and military. The industry in Poznań is dominated by electrical engineering, chemical and food branches. The area examined in this study is under constant influence of a variety of industrial and municipal emission sources, which mainly include the following: heat and power plants CFPP Karolin and CFPP Garbary, local boiler rooms and household furnaces, where coal is the primary fossil fuel. The other mercury sources include the following: municipal and hospital waste landfills, cement plants, sewage treatment plants, factories and plants that use high-temperature industrial processes and road traffic. The average annual temperature in Poznań is about 8.5 °C, and the prevailing wind direction is northwest. Poznań has the lowest annual precipitation in Poland (<550 mm), and a 32 % decline in precipitation occurs during the winter season. The growing season for this area is one of the longest in Poland, from 200 to 220 days (RIEP 2012, 2013).

### Sampling and chemical analysis

Samples of buds, leaves (*Tp* and *Ap*) and soil were collected from the area of Poznań city in four periods of the growing season, from April to November 2013 (marked as IV, VI, VIII, XI). The research material came from 22 points located in Poznań. These sampling points were chosen from the areas where both tree species grow close to each other. Additionally, soil samples were collected from the same points. The areas where samples (buds, leaves, soil) were collected are presented in Fig. 1, and sampling points located in the city centre are in the circle.

Soil samples were collected from a 20-cm layer of soil, using the soil sampler, and placed in PE containers. Visible plant parts and stones were removed from the samples. Bud samples were collected in April (IV), and growing leaves—from June (VI) to August (VIII)—from a height of 1.5 m above the ground. In November (IX), the leaves that fallen from the trees were collected. The plant material was placed in polyethylene bags. The geographical position of each sampling point was registered with a GPS navigation device Garmin 60 CSx (Table. 2). After delivery to the laboratory, the research material (soil, leaves and buds) was dried at room temperature, then homogenized in an agate mortar and sieved through a 0.15-mm mesh. The leaf samples were not washed. A 1.00 ± 0.01 g of each sample was weighted and placed in a digestion vessel made of PTFE. Then, 9 ml of HCl and 3 ml of HNO_3_ were added. The mixture was left for 12-h slow digestion. Subsequently, the samples were mineralized using a Mars 5 microwave digestion system, according to a modified EPA method no. 3051 (Frankowski et al. 2013). Acids of the highest purity (Sigma-Aldrich, USA), dedicated to the analysis of mercury, were used. Tin(II) chloride of low mercury content (Merck Darmstadt, Germany) was used for the preparation of the reduction solution. Deionized water was obtained from the Hydrolab System (Poland) and additionally purified from trace amounts of mercury with a stream of argon for 12 h. The calibration working solutions were prepared by appropriate dilution of the standard solution of 1000 ± 2 mg/l Hg(NO_3_)_2_ (Merck, Darmstadt, Germany) in 12 % HNO_3_. Mercury concentration in soil and plant samples was determined using a cold-vapour atomic fluorescence spectrometer (CV-AFS)—Millennium Merlin Analyzer 10.025 (PS Analytical, England). Similar analytical method for the determination of total mercury was applied by Boszke et al. (2008), Boszke and Kowalski (2006, 2007).

### CV-AFS method validation

Certified reference materials for soil, SRM 2709, and leaves, SRM 1515 (National Institute of Standards and Technology, USA), were used to verify the analytical method. The CRMs were analysed in ten replicates, and the average value, standard deviation and recovery of the method [%] were calculated. The results of the mercury content in CRM are presented in Table 1.

**Table 1:** Mercury concentration in the certified reference materials

		SRM 2709	SRM 1515
Certified value	[ng g^−1^]	1400 ± 80	44 ± 4
Determined value	[ng g^−1^]	1435 ± 34	43 ± 2
Recovery	[%]	102.5 ± 2.5	97.7 ± 4.5

## Results and discussion

**Fig. 2:**
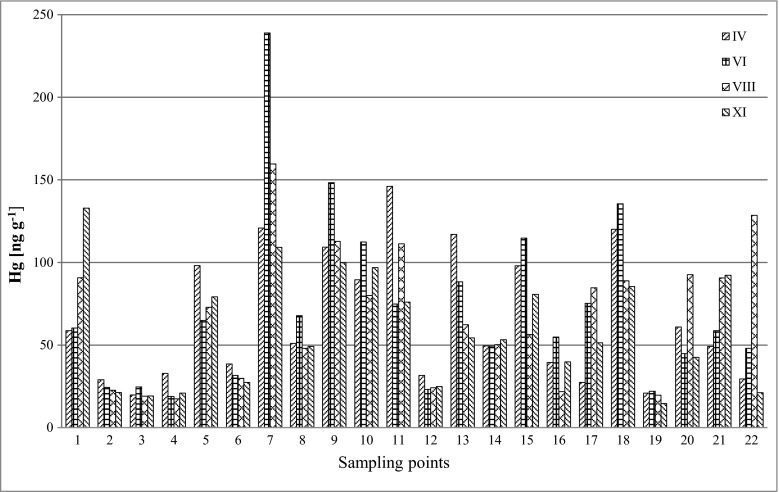
Concentration of mercury in soil samples (ng g^−1^)

**Fig. 3:**
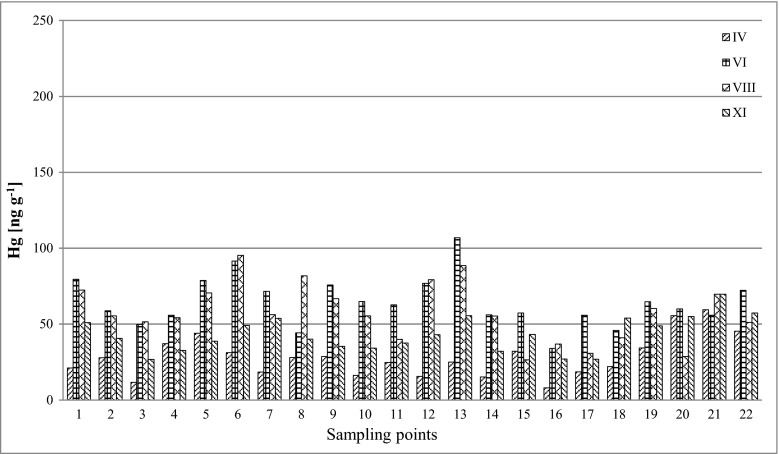
Concentration of mercury in buds and leaves of *Ap* (ng g^−1^)

**Fig. 4:**
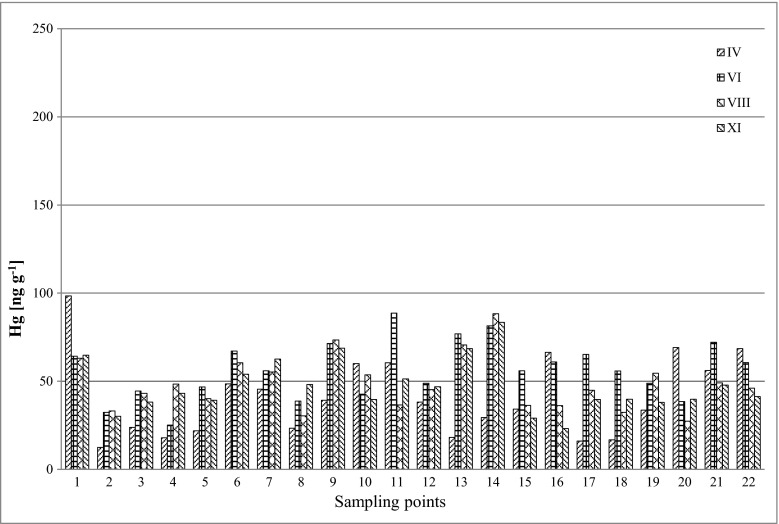
Concentration of mercury in buds and leaves of *Tp* (ng g^−1^)

The results of mercury concentration in the samples of soil and plants (*Ap* and *Tp*) collected from the Poznań area are presented in Table 2 and in Figs. 2, 3 and 4.

**Table 2:** Mercury concentration in the samples of soil, buds and leaves of *Ap* and *Tp* [ng g^−1^]

Sample	Coordinations		Concentration of mercury [ng g^−1^]											
			Soil				*Ap*				*Tp*			
			IV	VI	VIII	XI	IV	VI	VIII	XI	IV	VI	VIII	XI
1	N 52° 28′ 39.5″	E 16° 47′ 04.1″	58.7 ± 1.5	60.3 ± 1.2	90.7 ± 2.9	132.9 ± 3.5	98.4 ± 3.1	64.2 ± 1.7	63.1 ± 1.6	64.8 ± 1.7	21.1 ± 0.8	79.6 ± 2.2	72.4 ± 2.0	51.0 ± 1.3
2	N 52° 28′ 16.4″	E 16° 51′ 21.0″	29.0 ± 0.5	24.4 ± 0.3	22.6 ± 0.5	21.3 ± 0.5	12.4 ± 0.5	32.3 ± 0.9	33.1 ± 0.9	30.1 ± 0.9	27.8 ± 0.9	58.8 ± 1.5	55.4 ± 1.9	40.7 ± 1.0
3	N 52° 28′ 23.4″	E 16° 48′ 12.4″	19.8 ± 0.3	24.6 ± 0.4	19.0 ± 0.6	19.1 ± 0.5	23.8 ± 0.6	44.4 ± 1.3	43.2 ± 1.4	38.1 ± 1.0	11.7 ± 0.6	49.8 ± 1.4	51.6 ± 1.5	26.7 ± 0.9
4	N 52° 26′ 09.4″	E 16° 50′ 34.6″	32.8 ± 0.7	18.9 ± 0.1	17.4 ± 0.5	20.9 ± 0.6	17.9 ± 0.4	25.1 ± 0.8	48.4 ± 1.5	43.2 ± 1.2	37.1 ± 1.0	55.9 ± 1.5	54.2 ± 1.5	32.7 ± 1.0
5	N 52° 24′ 54.5″	E 16° 52′ 57.4″	98.1 ± 2.9	64.9 ± 2.4	72.8 ± 2.8	79.2 ± 2.2	21.9 ± 0.6	46.8 ± 1.2	40.1 ± 1.0	39.2 ± 1.2	44.0 ± 1.2	78.8 ± 2.1	70.5 ± 1.9	38.8 ± 1.0
6	N 52° 27′ 28.8″	E 16° 54′ 48.6″	38.5 ± 1.0	31.5 ± 0.9	29.8 ± 0.8	27.3 ± 0.8	48.5 ± 1.2	67.1 ± 1.9	60.5 ± 1.6	54.0 ± 1.4	31.2 ± 1.0	91.5 ± 2,4	95.3 ± 2.5	49.1 ± 1.1
7	N 52° 27′ 03.9″	E 16° 56′ 34.5″	120.8 ± 2.9	238.9 ± 5.0	159.6 ± 5.2	109.1 ± 3.1	45.5 ± 1.3	56.0 ± 1.6	55.3 ± 1.5	62.6 ± 1.9	18.4 ± 0.8	71.6 ± 1.9	56.3 ± 1.4	53.9 ± 1.5
8	N 52° 26′ 27.9″	E 16° 55′ 23.4″	51.0 ± 1.1	67.8 ± 1.2	48.0 ± 1.6	49.1 ± 1.3	23.4 ± 0.9	38.7 ± 1.1	30.4 ± 0.9	48.2 ± 1.6	28.0 ± 0.9	44.3 ± 1.1	81.8 ± 2.2	40.1 ± 1.0
9	N 52° 25′ 49.0″	E 16° 56′ 35.5″	109.3 ± 3.0	148.3 ± 4.7	112.8 ± 3.4	99.8 ± 2.7	39.2 ± 1.1	71.4 ± 1.9	73.4 ± 1.9	68.7 ± 1.8	28.6 ± 0.9	75.8 ± 2.0	66.8 ± 1.7	35.4 ± 1.0
10	N 52° 24′ 40.1″	E 16° 55′ 06.7″	89.5 ± 2.7	112.4 ± 3.9	79.9 ± 2.2	96.8 ± 2.8	60.0 ± 1.8	42.7 ± 1.5	53.6 ± 1.4	39.7 ± 1.0	16.3 ± 0.5	64.9 ± 1.7	55.5 ± 1.5	34.2 ± 1.1
11	N 52° 24′ 02.0″	E 16° 55′ 54.0″	146.1 ± 4.3	74.8 ± 2.5	111.3 ± 3.7	75.9 ± 2.0	60.5 ± 1.9	88.7 ± 2.5	36.5 ± 1.0	51.3 ± 1.4	24.7 ± 0.8	62.7 ± 1.6	40.0 ± 1.2	37.5 ± 1.2
12	N 52° 24′ 24.3″	E 16° 50′ 14.9″	31.5 ± 0.7	23.1 ± 0.6	24.1 ± 0.5	24.9 ± 0.5	38.2 ± 1.2	48.8 ± 1.3	45.2 ± 1.4	46.9 ± 1.2	15.6 ± 0.6	76.9 ± 2.2	79.3 ± 2.5	43.0 ± 1.1
13	N 52° 24′ 05.5″	E 16° 52′ 24.2″	117.0 ± 3.1	88.2 ± 2.5	62.3 ± 1.9	54.3 ± 1.4	18.1 ± 0.7	76.9 ± 2.0	70.6 ± 2.0	68.5 ± 1.8	25.0 ± 0.8	106.9 ± 2,8	88.6 ± 2.5	55.6 ± 1.4
14	N 52° 23′ 16.8″	E 16° 51′ 40.5″	49.5 ± 1.5	49.3 ± 1.3	50.2 ± 1.1	53.2 ± 1.3	29.4 ± 0.9	81.4 ± 2.2	88.3 ± 2.3	83.4 ± 2.5	15.2 ± 0.6	56.1 ± 1.4	55.3 ± 1.4	32.1 ± 0.9
15	N 52° 24′ 29.7″	E 16° 56′ 50.7″	97.9 ± 2.0	114.8 ± 3.8	56.3 ± 1.5	80.7 ± 2.5	34.2 ± 0.9	55.9 ± 1.6	36.3 ± 1.1	29.1 ± 1.1	32.1 ± 0.9	57.3 ± 1.5	26.5 ± 0.9	43.2 ± 1.2
16	N 52° 22′ 36.4″	E 16° 57′ 40.5″	39.4 ± 0.7	54.8 ± 1.6	21.8 ± 0.5	39.8 ± 1.0	66.4 ± 1.9	60.9 ± 1.8	36.2 ± 1.0	23.1 ± 0.8	8.0 ± 0.3	34.1 ± 0.9	36.8 ± 1.3	27.0 ± 0.9
17	N 52° 22′ 18.0″	E 16° 59′ 42.8″	27.3 ± 0.3	75.2 ± 2.0	84.7 ± 2.3	51.3 ± 1.3	16.1 ± 0.6	65.3 ± 1.9	44.9 ± 1.2	39.7 ± 1.1	18.5 ± 0.7	55.9 ± 1.8	30.8 ± 0.9	26.9 ± 0.7
18	N 52° 21′ 05.7″	E 16° 01′ 30.4″	120.1 ± 3.5	135.5 ± 4.2	88.9 ± 2.5	85.4 ± 2.8	16.8 ± 0.5	55.8 ± 1.5	32.3 ± 0.9	39.8 ± 1.2	22.0 ± 0.7	45.9 ± 1.2	41.0 ± 1.1	54.0 ± 1.4
19	N 52° 19′ 55.4″	E 16° 55′ 27.4″	20.9 ± 0.2	22.0 ± 0.3	19.6 ± 0.6	14.5 ± 0.6	33.6 ± 0.8	48.9 ± 1.5	54.6 ± 1.4	38.0 ± 1.0	34.3 ± 1.0	64.7 ± 1.6	60.3 ± 1.5	48.9 ± 1.4
20	N 52° 18′ 38.2″	E 16° 58′ 39.4″	60.9 ± 1.0	44.7 ± 1.2	92.6 ± 2.7	42.5 ± 1.2	69.1 ± 2.0	38.5 ± 1.3	27.3 ± 0.8	39.8 ± 1.2	55.6 ± 1.5	60.0 ± 1.6	28.6 ± 0.9	55.0 ± 1.5
21	N 52° 25′ 29.1″	E 16° 59′ 38.4″	49.1 ± 1.8	58.7 ± 1.7	90.6 ± 2.7	92.2 ± 2.6	56.1 ± 1.7	72.1 ± 2.0	49.1 ± 1.4	47.8 ± 1.3	59.5 ± 1.7	55.9 ± 1.4	69.7 ± 1.9	69.7 ± 1.5
22	N 52° 26′ 42.0″	E 17° 00′ 33.0″	29.5 ± 0.8	48.0 ± 1.5	128.5 ± 3.6	21.1 ± 0.4	68.5 ± 2.0	60.6 ± 1.7	46.1 ± 1.4	41.3 ± 1.2	45.4 ± 1.3	72.3 ± 2.0	51.1 ± 1.4	57.4 ± 1.3

Based on the results of mercury concentrations in the samples of soil (*n* = 88), buds (*n* = 22) and leaves (*n* = 66) of the two tree species, the Shapiro-Wilk test was performed. At the significance level of *α* = 0.05, the following *p* values were obtained: 0.000008 for soil samples, 0.084 for buds of *Tp*, 0.197 for buds of *Ap*, 0.054 for leaves of *Tp* and 0.093 for leaves of *Ap*. It was found that the results of mercury in soil samples were not normally distributed. The *t* test for the samples of both tree species showed that there was no statistically significant differences between the mean values of Hg concentration only for the samples of linden leaves (VI–XI) and maple leaves (VI–XI) (*p* = 0.137) (Table 3). The Wilcoxon test showed that the mean value of mercury for soil samples, 65.8 ± 41.7 ng g^−1^ (median 55.6 ng g^−1^), was statistically different from the mean values obtained for *Tp* and *Ap* bud samples (IV), and *Tp* leaf samples (VI–XI), but not statistically different from *Ap* leaf samples (VI–XI) (*p* = 0.086) (Table 3).

**Table 3:** Statistical summary of mercury concentrations (ng g^−1^) in the samples

Sample	Average ± S.D.	Median	Minimum	Maximum
Soil (IV–XI)	65.8 ± 41.7	55.6	14.5	238.9
*Ap* tree buds (IV)	40.8 ± 22.7	36.2	12.4	98.7
*Ap* leaves (VI–IX)	50.7 ± 15.8	48.0	23.1	88.7
*Tp* tree buds (IV)	28.2 ± 13.6	26.6	8.0	59.5
*Tp* leaves (VI–IX)	55.4 ± 18.1	55.4	26.5	106.9

When analysing the results of soil samples (Table 2) in terms of the points of origin, it was found that the highest concentration of mercury was determined in a sample collected at point no. 7 in June (VI), and it equaled 238 ng g^−1^. Throughout the whole study period (four sampling campaigns, from April to November), that point was also characterized by the highest average mercury content in the soil—157.1 ng g^−1^ (median 140.0 ng g^−1^). Lower average Hg concentrations were determined for point nos. 9—117.6 ng g^−1^ (median 111.1 ng g^−1^), 18—107.5 ng g^−1^ (median 140.0 ng g^−1^) and 11—102.0 ng g^−1^ (median 93.6 ng g^−1^). The lowest average mercury concentrations (19.3–31.8 ng g^−1^) for four periods of research were obtained for point nos. 19, 3, 4, 2, 12 and 6, located on the outskirts of Poznań. The places where the lowest levels of mercury in soils were measured are not well populated and can be described as the areas with low-density development and small network of roads, mainly local access roads. In contrast, the highest concentrations of mercury in soil were determined in the samples collected in the centre of Poznań (Fig. 1). The results of the Mann-Whitney *U* test showed that the mean value of mercury in the soil samples taken from the Poznań centre, for all the measurement periods (IV–XI), amounted to 95.5 ± 39.1 ng g^−1^ (median 91.4 ng g^−1^) and was statistically different from the mean value of mercury for the other soil samples—48.8 ± 32.9 ng g^−1^ (median 39.0 ng g^−1^). Mercury content of soils from the centre of Poznań was two times higher than that from other parts of the city. Pasieczna (2012) concluded that mercury concentrations in soils located in the centre of a city are four times higher than those in samples collected on the outskirts. Tijhuis et al. (2002) measured the average mercury content of soils taken from the centre of Oslo to be 480 ng g^−1^, which was over eight times higher than in other parts of the city. The average mercury content of soil samples collected from central Stockholm (860 ng g^−1^) was 50 times higher than the content of rural soils (arable land) collected from the vicinity of Stockholm city (Linde et al. 2001). The city centre of Poznań can be characterized by a very compact way of building and large network of narrow low-capacity streets which are often congested. Therefore, high mercury concentrations in soils of this area should be mainly linked to the effect of mercury emission to the air, from the combustion of fossil fuels in domestic furnaces during the heating season, liquid fuels consumed by cars and the re-emission from the air to soil. Taking into account the fact that the highest mercury concentrations were determined at point nos. 7, 9 and 11, located very close to the main emitters for Poznań city, i.e. CFPP Garbary and Karolin, it can be assumed that these power plants have a major negative impact on Hg content of soils. The prevailing wind direction, i.e. north-western, suggests that other potential mercury emitters, e.g. municipal landfill, airports and motorways, are of minor importance in this regard.

During the spatial analysis of the results of average mercury content of *Tp* and *Ap* samples in four measurement periods (April–November), it was observed that the highest concentrations of mercury in the leaves of both tree species were not found for the same sampling points. The highest average Hg levels in *Tp* samples were determined at point nos. 1 and 14, respectively [72.6 ng g^−1^ (median 64.8 ng g^−1^) and 70.6 ng g^−1^ (median 81.4 ng g^−1^)], while the highest average Hg values for *Ap* were measured at point nos. 6 and 8, respectively [69.6 ng g^−1^ (median 72.1 ng g^−1^) and 66.8 ng g^−1^ (median 70.3 ng g^−1^)]. It was also observed that the points were the highest average mercury concentrations in *Ap* and *Tp* were measured did not coincide with the points of the highest concentrations in soils. The results of mercury concentration in 22 samples collected in four measurement periods (IV–XI) are shown in Table 4.

**Table 4:** Statistical summary of mercury concentrations (ng g^−1^) in the examined samples, for the four research periods (IV–XI)

Sample	Average ± S.D.	Median
Soil IV	65.3 ± 39.3	50.2
Soil VI	71.9 ± 52.5	59.5
Soil VIII	67.4 ± 40.4	67.5
Soil XI	58.7 ± 34.4	52.3
*Ap* IV	40.8 ± 22.7	36.2
*Ap* VI	56.5 ± 16.3	55.9
*Ap* VIII	48.6 ± 15.4	45.7
*Ap* IX	47.2 ± 14.7	42.3
*Tp* IV	28.2 ± 13.5	26.4
*Tp* VI	64.5 ± 16.4	61.4
*Tp* VIII	57.6 ± 19.2	55.5
*Tp* IX	43.3 ± 11.5	41.9

The non-parametric Wilcoxon signed-rank test was selected to compare the obtained average values of mercury concentration in the samples of soil, buds and leaves of both tree species, collected in IV and VI (Table 4). For samples collected in the other periods, the *t* test was performed. Based on the statistical analysis, it was found that the average values of mercury were statistically different only for the following months: April (IV)—for *Ap* and *Tp* (*p* = 0.0304), and soil and *Tp* (*p* = 0.0362), June (VI)—for soil and *Ap* (*p* = 0.011), August (VIII)—for soil and *Tp* (*p* = 0.00472) and November (XI)—for soil and *Ap* (*p* = 0.0445). The analysis of the correlation coefficient between the values of mercury content in different types of samples for all the measurement periods (IV–XI) was also conducted. For samples collected in April (IV) and June (VI), the Spearman correlation was used, while the Pearson correlation was used for the other samples (Table 5).

**Table 5:** Correlation coefficients between the values of mercury content in soil, linden and maple samples collected in different research periods

	Soil IV	Soil VI	Linden IV	Linden VI	Maple IV	Maple VI
Soil IV^a^	1.000000	0.774139	0.174478	0.303219	0.025409	0.239118
Soil VI^a^	0.774139	1.000000	0.024280	0.373235	−0.155280	0.057094
Soil VIII^b^	1.000000	0.659564	0.085055	0.335306	−0.183204	0.412237
Soil XI^b^	0.659564	1.000000	0.213842	0.367927	−0.004918	0.226696

^a^Spearman correlation coefficient

^b^Pearson correlation coefficients

**Fig. 5:**
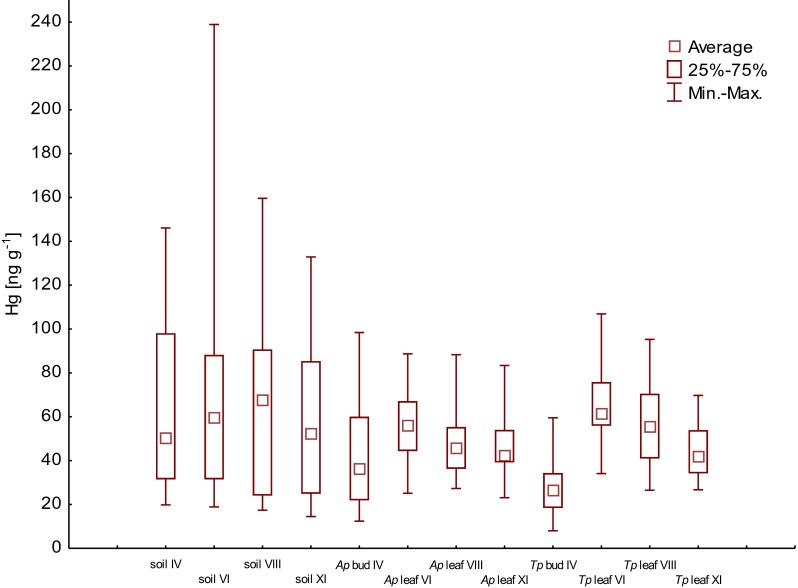
Average mercury concentrations (ng g^−1^) in the samples of soil, *Ap* and *Tp*, in different measurement periods

The performed statistical calculations (at *p* = 0.05) showed no correlation between the concentrations of mercury in soil, buds and leaves in the same periods of measurements. Similar results (lack of correlations) were obtained by e.g. Pérez-Sanz et al. 2012; Lodenius 2013; Amorós et al. 2014. When analysing average Hg values for each type of samples taken in four periods (IV—XI), the variability of mercury concentrations in time was observed (Fig. 5).

Similar relationships related to the variability of mercury concentrations in time were found for leaf samples by Ericksen et al. (2003), Poissant et al. (2008), Rutter et al. (2011) and Tabatchnick et al. (2012). The distribution of average Hg concentrations for samples of *Ap*, *Tp* and soil was similar for each of the measurement months. The maximum average concentrations of mercury were found in June (VI), and in the following months, the concentrations decreased. When analysing average mercury concentrations in soil and tree samples, it was observed that the average value of Hg for soils in November (XI) (58.7 ng g^−1^) was lower than the value determined in April (IV) (65.3 ng g^−1^). Such situation can be explained by the accumulation of mercury, originating mainly from snow and dry deposition (associated with the heating season), in the surface layers of soil in winter. The frozen soil stops or reduces the infiltration of water with pollutants into the deeper layers. Then, the sorption of mercury occurs in the uppermost soil layers which are rich in organic matter. The increase in the concentration of mercury in soils from April (IV) to June (VI) was probably related to the mercury supply from the atmosphere and mercury release after organic matter mineralization, e.g. from leaves, during the period of temperature increase in spring. Based on temperature measurements made by the Regional Inspectorate for Environmental Protection in Poznań, it was observed that during the heating season (December 2012—March 2013) preceding the study period, the temperature pattern was as follows: very cold December, moderately cold January and February and extremely cold March. In March, the minimum daily average temperature was −6 °C, while in April, a significant increase in the temperature was registered, which resulted in the rapid growth of vegetation (RIEP 2012, 2013). The probable reason for the highest concentrations of mercury in leaves, determined in June, was the fact that the plants uptake minerals, including mercury, from soil, and this process is the most intensive in the initial vegetative phase. Additionally, gaseous mercury can be assimilated into the plant system from the atmosphere through the stomata of leaves, and mercury bound to particulate matter can be adsorbed on the surface of leaves. The exchange of mercury through leaf stomata is less intensive at the end of the growing season, which is related to the ageing of the leaves (Poissant et al. 2008). It is believed that mercury content of plant roots corresponds to the content of soils. About 95–99 % of mercury uptaken by the root system remains in the roots, and only a small portion can be transported to the leaves (Ericksen and Gustin 2004). Bishop et al. (1998) reported that this transport equals up to 11 %. The value is primarily influenced by mercury form, the pH of soil, organic matter content and plant genotype (Alloway and Ayres 1999; Kabata-Pendias 2001). The relationship between mercury concentration in the plant and soil can be described by the transfer coefficient (metal concentration in the above-ground part of a plant divided by the concentration of this metal in the soil), proposed by Kloke et al. (1984), which is in a range of 0.01–0.1 for mercury. In the case of the analysed samples, the average transfer coefficient was 1.04 (median 0.82, range 0.14–2.79) for *Tp* and 1.11 (median 0.77, range 0.15–3.37) for *Ap*. In the following sampling periods, the coefficient equaled: IV—0.88, VI—1.11, VIII—1.11 and XI—1.12 in linden, and IV—0.61, VI—1.38, VIII—1.38 and XI—1.09 in maple. Such high values indicated that the main source of mercury in the examined plants was the particulate matter adsorbed on the leaves of both tree species (leaves were not washed for the study) and the gas exchange through the stomata followed by the accumulation in leaves. These conclusions are similar to those proposed by other researchers. Fay and Gustin (2007) found that the increase in mercury concentration in leaves is primarily caused by mercury in the atmosphere. They observed that the variability in the concentrations of mercury in the air, from 3.1 to 30.1 ng m^−3^, accompanied by constant Hg concentrations in the soil (60 ng g^−1^), was followed by a 3.8-fold and 3.1-fold increase in mercury concentrations in the leaves of *Juniperus scopulorum* and *Robini pseudoacaci*, respectively. When the mercury concentration in soil was increased from 60 to 27,700 ng g^−1^, the increase in leaves was only 1.19-fold for *J. scopulorum* and 1.01-fold for *R. pseudoacaci* (Fay and Gustin 2007). Ericksen and Gustin (2004) observed similar dependencies on the basis of the results of *Populus tremuloides* investigations. When comparing the average mercury concentrations obtained in the present study for the soil from Poznań area with the results of previous studies carried out for the same area (Table 6), it can be noticed that the mercury concentrations presented in this paper are lower than the results obtained in previous years (Falandysz et al. 1996; Lis and Pasieczna 2005; Boszke and Kowalski 2006; Frankowski et al. 2007; Kowalski et al. 2012). In the case of soil, it was found that they are also lower than the concentrations of mercury in other large Polish cities, i.e. Katowice, Warsaw, Cracow and Gdynia (Pasieczna 2012). When comparing the results of mercury in the leaves of the two tree species from Poznań with the literature data (Table 6), it was noticed that the concentrations have changed in the period of 8 years (Frankowski et al. 2007; Kowalski et al., 2012). The present study shows a decline in mercury concentrations in leaves; however, they are still much higher than the values determined for Oborniki town (Frankowski et al. 2007), which is located about 40 km north of Poznań, at the edge of the Noteć Forest—one of the largest and least populated forest compounds in Poland. The average concentration of mercury in the *Ap* and *Tp* leaf samples from the Noteć Forest was 8.4 ng g^−1^ (6.8–9.2 ng g^−1^) and 10.9 ng g^−1^ (10.0–11.4 ng g^−1^), respectively. Poissant et al. (2008) also measured lower concentrations of Hg in the leaves of *Acer saccharum Marsh* collected from forest areas, and the values were from 8.7 ± 1.5 to 30.8 ± 3.0 ng g^−1^ for different vegetative periods. Based on the study results from the years 2005–2013, a tendency for high mercury concentrations could be observed for plant samples taken from the sampling point no. 1 (northern edge of Poznań). In those years, the maximum or the highest values were determined at that point. The probable reason for such concentrations was a local emission source which introduced mercury into the atmosphere.

**Table 6:** Comparison of total mercury concentration (ng g^−1^) in soil and leaf samples determined in different studies

Study area	Type of sample	(ng g^−1^) dry weight	Reference
Poznań, Poland	Soil	Average 65.8; median 55.6 (range 14.5–238)	This study
Poznań, Poland	Soil	Average 96	Falandysz et al. 1996
Poznań, Poland	Urban soil of Poznań	Average 3120, median <50 (range <50–712,000)	Lis and Pasieczna 2005
Poznań Poland	Soil	146 ± 130 (range 17–746)	Boszke and Kowalski 2006
Poznań, Poland	Soil	Average 132 (47–212)	Kowalski et al. 2012
Katowice Poland	Soil	(Range 80–7550)	Pasieczna 2012
Cracow, Poland	Soil	(Range <50–1380)	Pasieczna 2012
Warsaw Poland	Soil	(Range <50–10,780)	Pasieczna 2012
Gdynia, Poland	Soil	Average 60 (range 60–300)	Pasieczna 2012
Poznań, Poland	Large-leaved linden	Average 48.4 (range 12.4–98.4)	This study
Poznań, Poland	Norway maple	Average 48.3 (range 8.0–106.9)	This study
Poznań, Poland	Large-leaved linden	Average 123 (range 10–544)	Frankowski et al. 2007
Poznań, Poland	Norway maple	Average 89.8 (range 6.6–328)	Frankowski et al. 2007
Oborniki, Poland	Large-leaved linden	Average 10.9 (range 10.0–11.4)	Frankowski et al. 2007
Oborniki, Poland	Norway maple	Average 8.4 (range 6.8–9.2)	Frankowski et al. 2007
Poznań, Poland	Large-leaved linden	Average 165 (range 96–233)	Kowalski et al. 2012
Poznań, Poland	Norway maple	Average 162 (range 116–207)	Kowalski et al. 2012

The recent decrease in mercury concentrations in soil and leaf samples from Poznań was the result of continuous improvements in air quality. According to data from the Central Statistical Office (RIEP 2012, 2013), a decrease in the emissions of particulate matter and gaseous pollutants, mainly from the heat and power plants in Poznań, including CFPP Karolin and CFPP Garbary, has been observed recently in the Poznań Agglomeration (Environmental Protection Programme 2013). Due to large emissions of PM, its largest emitters—CFPPs in Poznań—changed the technology of ash removal and stopped using boilers which were the most burdensome for the environment. The so-called low-stack emission was also decreased as a result of investments aimed at the conversion of the way of heating, from coal-fired domestic furnaces/boilers into central heating from CFPPs, or the replacement of coal-fired domestic boilers/furnaces, often in very poor condition, for modern gas- or liquid-fueled boilers which are more environment friendly. The increase of environmental awareness among Poles also contributed to the improvement of the quality of the air. Until now, it was common for many people to burn wastes such as cardboards, newspapers and magazines, PET bottles, rubber and textiles in their household boilers/furnaces, especially at the beginning and end of the heating season. Currently, as a result of public information campaigns and mandatory segregation of waste, many items are recycled or go to a landfill, instead of being burnt. Other investments which are more often carried out to reduce the air pollution include alternative energy sources, e.g. solar panels in public buildings, thermo-modernization of buildings, introduction of new technologies and installation of industrial dust collectors.

## Conclusions

Based on the investigations of soil, bud and leaf samples of *Ap* and *Tp*, carried out in four periods of the growing season, from April (IV) to November (XI), the following conclusions were drawn:

1. Mercury concentrations in 88 samples of soil were statistically significantly higher than the values in buds and leaves collected from the area of Poznań. The statistical analysis of buds and leaves of the two tree species revealed that only for the leaf samples of *Tp* and *Ap* there were no significant differences between the average concentrations of mercury.
2. The analysis of the spatial variability in mercury content of soils showed a twofold higher concentrations of mercury in the city centre than in other parts of the city. There was no similar dependence observed for tree samples. Higher concentrations in soils were primarily caused by the so-called low-stack emission from the combustion of fossil fuels in domestic furnaces/boilers during the heating season and by the emission from liquid fuels combusted by cars. A negative impact of the two power and heat plants in Poznań, CFPP Garbary and CFPP Karolin, was also reported.
3. The variability of mercury concentration in soils in different periods of the growing season, from April (IV) to November (XI), was observed. In the first two periods of measurements (IV, VI), mercury content was higher, while in the following periods, the accumulation of mercury both in soil and leaf samples was lower. There was no correlation between mercury concentrations in leaves and mercury concentration in soils for any of the four measurement periods (April–November).
4. Based on the analysis of the transfer coefficient, it was found that high concentrations of mercury in the examined trees were the result of mercury coming from the particulate matter adsorbed on leaves of both tree species, mercury associated with gas exchange through the stomata and its further accumulation in leaves.
5. When comparing the past and current results of mercury concentration measured in soil and plant samples, it was noted that the values for the area of Poznań have decreased recently. This is mainly the effect of the air quality improvements in Poznań, after the introduction of ecological policies.
